# The complete chloroplast genome of *Mimusops elengi* (Sapotaceae: Sapoteae)

**DOI:** 10.1080/23802359.2022.2087550

**Published:** 2022-06-28

**Authors:** Qiyi Huang, Lijuan He

**Affiliations:** a School of Civil Engineering, Xiamen University Tan Kah Kee College, Fujian, China; b Xiamen Overseas Chinese Subtropical Plant Introduction Garden, Plant Introduction & Quarantine and Plant Product Key Laboratory of Xiamen City, Fujian, China

**Keywords:** *Mimusops elengi*, chloroplast genome, phylogenetic analysis

## Abstract

The first complete chloroplast genome sequences of *Mimusops elengi* Linaeus, 1753 (Sapotaceae: Sapoteae) were reported in this study. The cpDNA of *M. elengi* is 159,719 bp in length, contains a large single-copy region (LSC) of 88,935 bp and a small single-copy region (SSC) of 18,606 bp, which were separated by a pair of inverted repeat (IR) regions of 26,089 bp. The genome contains 132 genes, including 87 protein-coding genes, 8 ribosomal RNA genes, and 37 transfer RNA genes. The overall GC content of the whole genome is 36.8%. Phylogenetic analysis of 8 chloroplast genomes within the tribe Sapoteae suggests that the sister relationship of *Autranella* and *Tieghemella* are strongly supported. *Minusops* genus is close to Autranella and *Tieghemella*, although the support value is still low.

*Mimusops elengi* Linaeus, 1753 belongs to the tribe Sapoteae of Sapotaceae family, a. It is native to South and Southeast Asia and northern Australia, and has been known for its myriad values. Its timber is hard and durable. Its flower is aromatic. Its fruit is edible. It is also widely used in traditional medicine (Gupta 2013). It was introduced in China in 1962 from India and now is sparsely cultivated in Fujian, Guangdong, Guagnxi, Hainan and Yunnan (Bai et al. 2011). This tribe includes 12 genera. But its taxomomy and phylogenetic relationship remained controversial due to low resolution of limited molecular evidence (Swenson and Anderberg 2005; Kümpers et al. 2016). Till now, 8 plastomes representative of 7 genera from this tribe have been reported. Therefore, we sequenced the complete chloroplast genome of *M. elengi.* It cp genome sequence would contribute to the further study of this speices and also provide genetic information for the construction of the phylogenetic relationship among Sapoteae.

The silica-dried material of *M. elengi* was obtained and from Xiamen Overseas Subtropical Plant Introduction Garden, Fujian province in China (latitude 24.4485°N and longitude 118.0717°E). The specimens were deposited in its herbarium, OSBG with specimen code YZY20170264 (Mrs. He, helj2013@126.com). Total genomic DNA was extracted using Tiangen Plant Genomic DNA Kit (Tiangen Biotech Co., Beijing, China) and sheared into ca. 350 bp fragments. Libraries for paired-end (PE) Illumina sequencing were conducted following the standard protocol of manufacture (TruSeq Library Construction Kit) and sequenced from both ends of 150 bp fragments on the Illumina HiSeq 2000 platform at Novogene Co., Ltd (Beijing, China). The complete chloroplast genome was assembled, circulared and annotated using GENIOUS PRIME 2019.1.1 (Kearse et al. 2012) with cp genome of *M. coriacea* (MW846242) as reference. The chloroplast genome was submitted to GenBank (http://www.ncbi.nlm.nih.gov/) with accession number OK458682.

Chloroplast genome size of *M. elengi* is 159,719 bp with the GC content of 36.8%. It consists of the traditional quadripartite structure: a large single-copy region of 88,935 bp, a small single copy region of 18,606 bp, and two inverted repeat regions of 26,089 bp. The new sequence possesses a total of 130 genes, containing 85 protein-coding genes (PCGs), 8 ribosomal RNA (rRNA) genes, and 37 transfer RNA (tRNA) genes.

In previous studies, the intrafamily relationship within Sapotaceae is difficult to resolve with limited markers (Swenson and Anderberg 2005). To achieve a reliable phylogenetic relationship, maximum likelihood tree was constrcuted based on 78 single copy protein-coding genes with 12 other Sapotaceae species using RAxML HPC v.8.2.4 (Stamatakis 2014). GTR + I + G model was selected as the best-fitting model determined by jModelTest v.2.1.1. *M. elengi* is revealed to be close to *Autranella* and *Tieghemella*, although the support value is still low (Figure 1). The sister relationship of the latter two genera are strongly supported. Our study shows the high strength of complete chloroplast genomes in uncovering the deep relationship within Sapotaceae.

**Figure 1.**
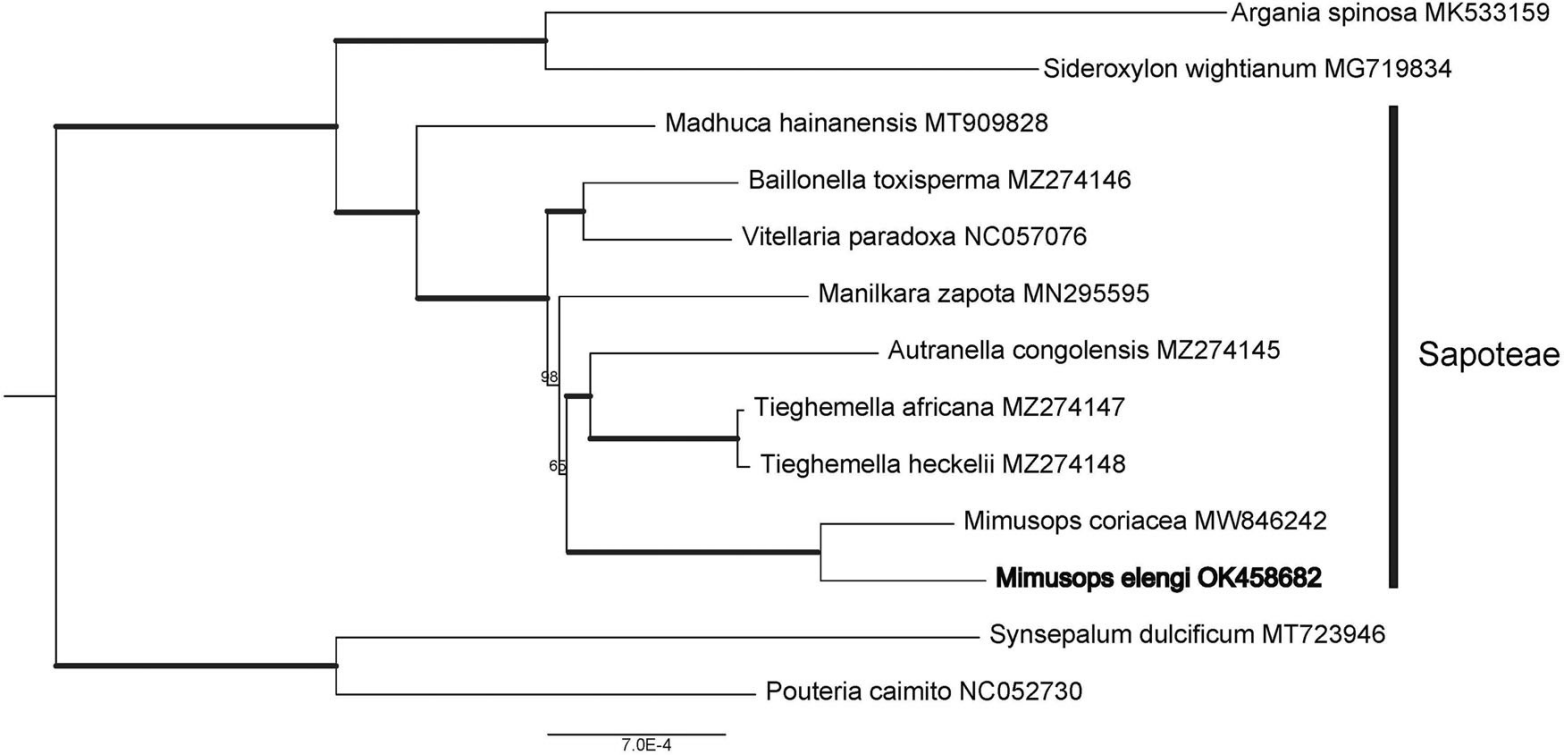
Maximum likelihood tree of *M. elengi* based on concatenated protein sequences of 78 protein-coding sequences shared by 13 Sapotaceae species. Numbers at the nodes indicates bootstrap values (1000 replicates).

## Data Availability

The genome sequence data that support the findings of this study are openly available in GenBank of NCBI at https://www.ncbi.nlm.nih.gov/nuccore/OK458682 under the accession no. OK458682. The associated BioProject, SRA, and BioSample numbers are PRJEB49293, ERS8701945, and SAMEA11052235 respectively.
